# Formation of a nucleoplasmic reticulum requires *de novo* assembly of nascent phospholipids and shows preferential incorporation of nascent lamins

**DOI:** 10.1038/s41598-017-07614-w

**Published:** 2017-08-07

**Authors:** Marek M. Drozdz, Haibo Jiang, Lior Pytowski, Chris Grovenor, David J. Vaux

**Affiliations:** 1 Sir William Dunn School of Pathology, University of Oxford, Oxford, OX1 3RE United Kingdom; 2 Centre for Microscopy, Characterisation and Analysis, The University of Western Australia, 35 Stirling Highway, Crawley, WA 6009 Australia; 3 Department of Materials, University of Oxford, Oxford, OX1 3PH United Kingdom

## Abstract

Structure of interphase cell nuclei remains dynamic and can undergo various changes of shape and organisation, in health and disease. The double-membraned envelope that separates nuclear genetic material from the rest of the cell frequently includes deep, branching tubular invaginations that form a dynamic nucleoplasmic reticulum (NR). This study addresses mechanisms by which NR can form in interphase nuclei. We present a combination of Nanoscale Secondary Ion Mass Spectrometry (NanoSIMS) approach and light microscopy techniques to follow formation of NR by using pulse-chase experiments to examine protein and lipid delivery to nascent NR in cultured cells. Lamina protein incorporation was assessed using precursor accumulation (for lamin A) or a MAPLE3 photoconvertible tag (for lamin B1) and membrane phospholipid incorporation using stable isotope labelling with deuterated precursors followed by high resolution NanoSIMS. In all three cases, nascent molecules were selectively incorporated into newly forming NR tubules; thus strongly suggesting that NR formation is a regulated process involving a focal assembly machine, rather than simple physical perturbation of a pre-existing nuclear envelope.

## Introduction

The nuclear envelope (NE) is a unique structure forming a physical barrier between the nuclear environment and the cytoplasm. It is comprised of two phospholipid bilayers, the inner nuclear membrane (INM) and outer nuclear membrane (ONM), with an intervening luminal space between them called the perinuclear space, and underlain by a lamin-rich proteinaceous meshwork^1^. Cell nuclei come in various shapes and can undergo extensive structural changes in response to external stimuli, in result of differentiation, replication, and cell migration or in pathological situations such as ageing or cancer. Our understanding of the origin of nuclear component required for such shape alterations is limited, especially in regard to tracking nascent phospholipid addition to nuclear membranes.

**Figure 1:**
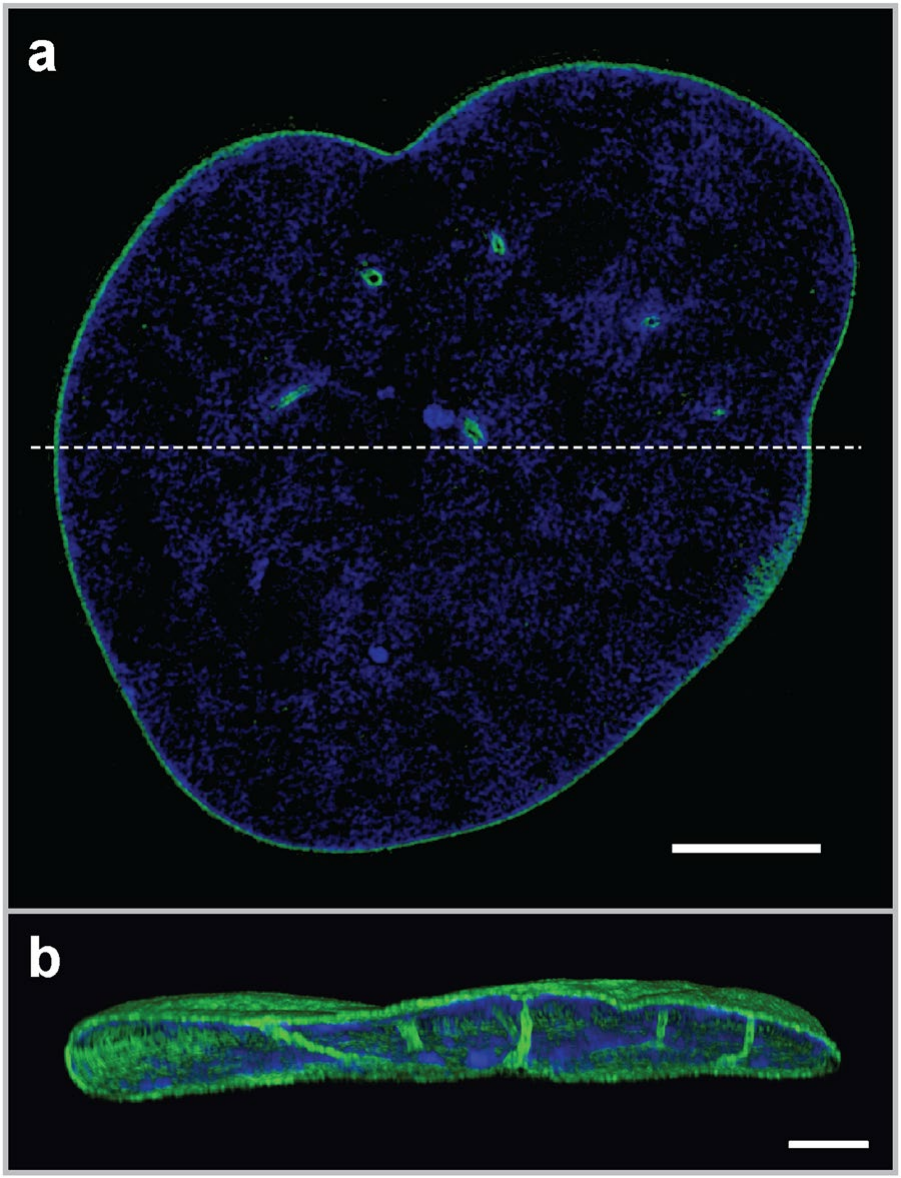
Nucleoplasmic Reticulum (NR) in normal Human Dermal Fibroblasts (HDFs). Super resolution light microscopy on untreated normal HDFs showing distribution of Lamin B1 (Green; anti-lamin B1 immunostaining) and chromatin (Blue: Di-amino-phenyl-indole; DAPI). (**a**) Nuclear mid-section showing intranuclear lamin B1 foci surrounding NR channels. (**b**) 3D reconstruction of nucleus with vertical cross-section (along dashed line shown in a) showing NR channels traversing the nucleus. Scale bar 2 µm.

An example of dynamic alterations to the NE is formation of a network of penetrating and branching invaginations, collectively referred to as the nucleoplasmic reticulum (NR)^2^. The NR structures can be composed of the INM only with perinuclear space core (type I NR) or both INM and ONM forming channels within the nucleus (type II NR) with cytoplasmic core^3^. Type II NR can be readily visualised by anti-lamin B immunostaining (Fig. 1). The NR is a widespread feature of many cells and tissues *in vitro* and *ex vivo*, both under normal cellular conditions^2, 4, 5^ and in pathological states such as cancer, Alzheimer’s Disease or ageing^6–8^. However, the exact function of the NR remains incompletely understood. It may play a role in the structural support of the nucleus and in communication between the cytoplasm and nucleoplasm by increasing the interface between these two environments. Moreover, there is tantalizing evidence implicating the NR in calcium signalling in sub-nuclear regions^9^. This contributes to regulation of gene expression and cell growth^10–12^, lipid metabolism^13^, and nuclear import-export. It is also suggested that the presence of NPC proteins and structures that resemble NPC at NR invaginations may impact chromatin organization^14^. Despite the ubiquity of NR structures, little is known about their formation, although time-lapse imaging shows that NR may form in interphase nuclei without the intervention of mitosis, which excludes several models for NR synthesis^14^.

Our objective was to devise a comprehensive approach for tracking of nascent population of nuclear envelope components, both protein and membrane phospholipids, and apply the techniques to study the changes of NE structure, in particular NR formation. We used protein tagging with photoconvertible MAPLE3 tag^15^ in combination with immunolabelling followed by light microscopy to analyse nascent components of nuclear lamina, whereas addition of new membranes to the nucleus was analysed by adapting high resolution Nanoscale Secondary Ion Mass Spectrometry (NanoSIMS) approach^16^ to biological samples.

## Results

### Light super resolution microscopy imaging shows nascent prelamin A at newly formed NR

It has been shown that nuclear morphology is affected, and NR complexity increased, upon prelamin A accumulation in interphase nuclei^14^. Similar changes have also been observed in Hutchinson-Gilford progeria syndrome (HGPS) cells and in physiological ageing^17–19^. This is likely an effect of the durable incorporation into the NE of lamin A variants harbouring a persistently farnesylated tail. Since prelamin A accumulation increases NR complexity, we first investigated whether prelamin A is incorporated into the nuclear lamina randomly or perhaps at specific deposition sites associated with nascent NR.

**Figure 2:**
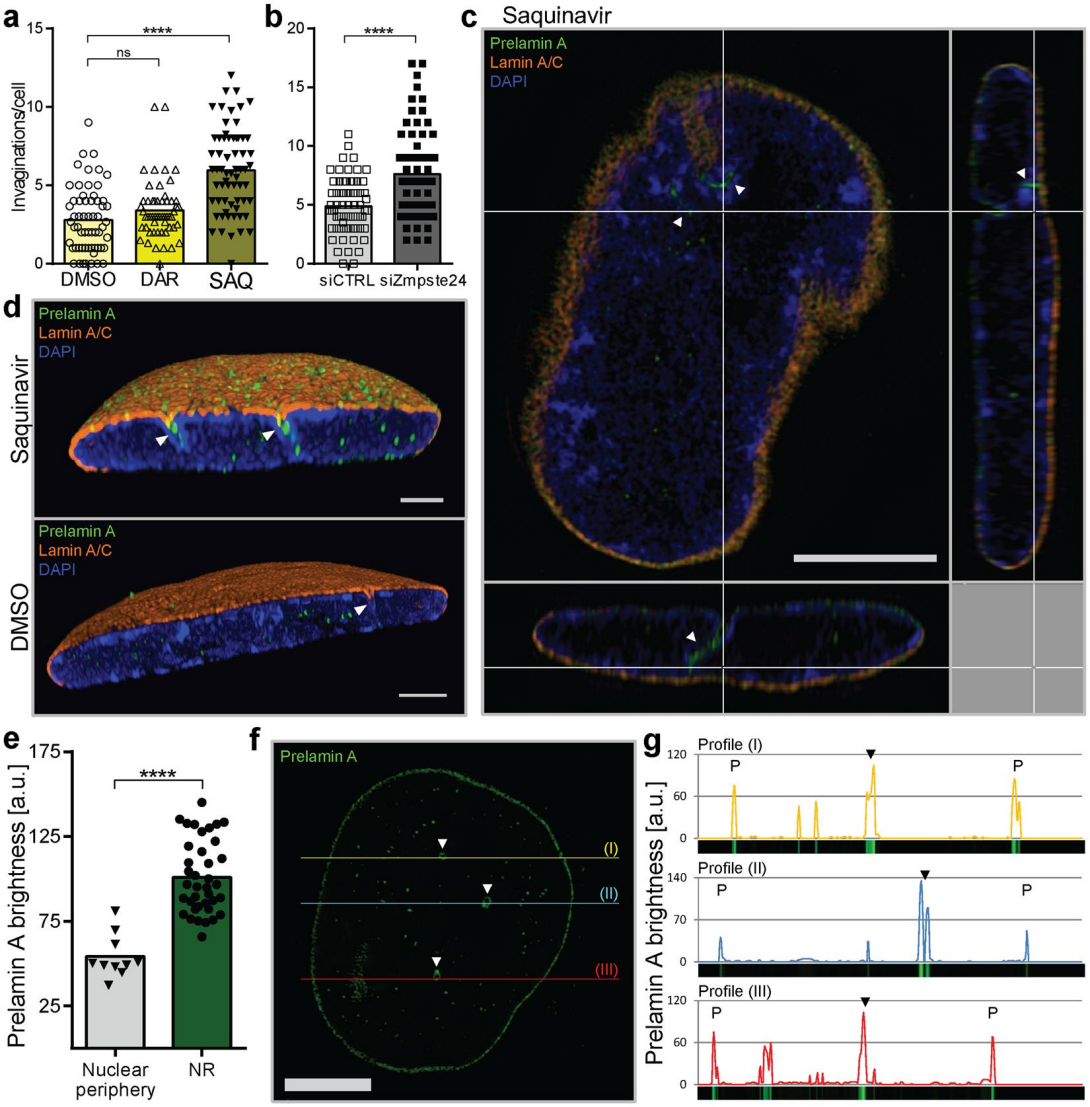
NR formation in response to prelamin A accumulation. Mean frequency of NR tubules in cells accumulating prelamin A under saquinavir treatment (SAQ) (**a**) and Zmpste24 siRNA knock-down (siZmpste24) (**b**) with corresponding controls: DMSO (vehicle), DAR (darunavir), and control siRNA (siCTRL); results from four independent experiments, 30 nuclei each. (**c**) Super resolution light microscopy on saquinavir-treated early passage HDFs with arrowheads pointing towards NR channels; scale bar, 5 µm. (**d**) 3D reconstruction of super resolution light microscopy data of cells treated with either saquinavir or DMSO; scale bar, 2 µm. (**e**) Quantification of prelamin A signal at nuclear periphery and NR channels; n = 10 nuclei. (**f**) An example of mid-section showing prelamin A immunostaining with arrowheads pointing towards NR channels; scale bar, 5 µm. (**g**) Prelamin A brightness along the color-coded profiles drawn in (**f**); black arrowheads correspond to NR channels, while “P” indicates nuclear periphery region. ****p < 0.0001; ns, p not significant.

Early passage normal Human Dermal Fibroblasts (HDFs) were cultured for 48 hours in the presence of saquinavir, an HIV proteinase inhibitor that also blocks Zmpste24 (also known as FACE1), the enzyme responsible for prelamin A proteolytic maturation^20^. In agreement with our previous work, saquinavir inhibition or specific siRNA knock-down of Zmpste24 leads to both prelamin A accumulation and increased occurrence of NR structures (Supplementary Fig. 1). Saquinavir treatment elicited a nearly 2-fold increase in the mean NR tubule number, while cells treated with vehicle control (DMSO) or with darunavir (another HIV proteinase inhibitor, but one that does not block Zmpste24^21^) remained unaffected (Fig. 2a). Furthermore, specific Zmpste24 knock-down with siRNA, revealed the same pattern of increased NR formation (Fig. 2b).

It is possible that saquinavir has an effect on proteolytic processes in the cell other than just inhibition of Zmpste24. Indeed, together with Hagen and colleagues, we observed that prolonged saquinavir treatment, apart from inducing NR, can also evoke apoptosis at least in some cell types^22^. However, in the current study of HDFs upon saquinavir treatment we did not observe vacuolation or evaginated plasma membrane blebs characteristic of apoptotic cells (Supplementary Fig. 2). Moreover, these cells treated with saquinavir for 48 hours, were later able to recover and re-normalise the NE morphology when the saquinavir was removed from the culture medium (Supplementary Fig. 3). Not only most of the prelamin A was processed to mature lamin A within 3 hours in saquinavir-free medium, but also within 24 hours the number of NR invaginations decreased to the level comparable to control cells, suggesting that NR is a dynamic structure. Moreover, reversibility of phenotypic changes and cell survival of the drug treatment is not consistent with saquinavir inducing apoptosis in these experimental settings. Given that prelamin A accumulation induces NR formation, in the next step super-resolution light microscopy was employed to investigate the distribution of prelamin A within the nuclear envelope following 48 hours of saquinavir treatment. This analysis revealed that prelamin A is highly enriched at the invagination sites (Fig. 2c,d) which are revealed as preferred foci of incorporation of new prelamin A copies (Fig. 2e,g), indicating that prelamin A is a building block for newly formed NR under these experimental conditions.

### Photoconvertible tagging of lamin B1 shows selective incorporation of the nascent protein into forming NR channels

**Figure 3:**
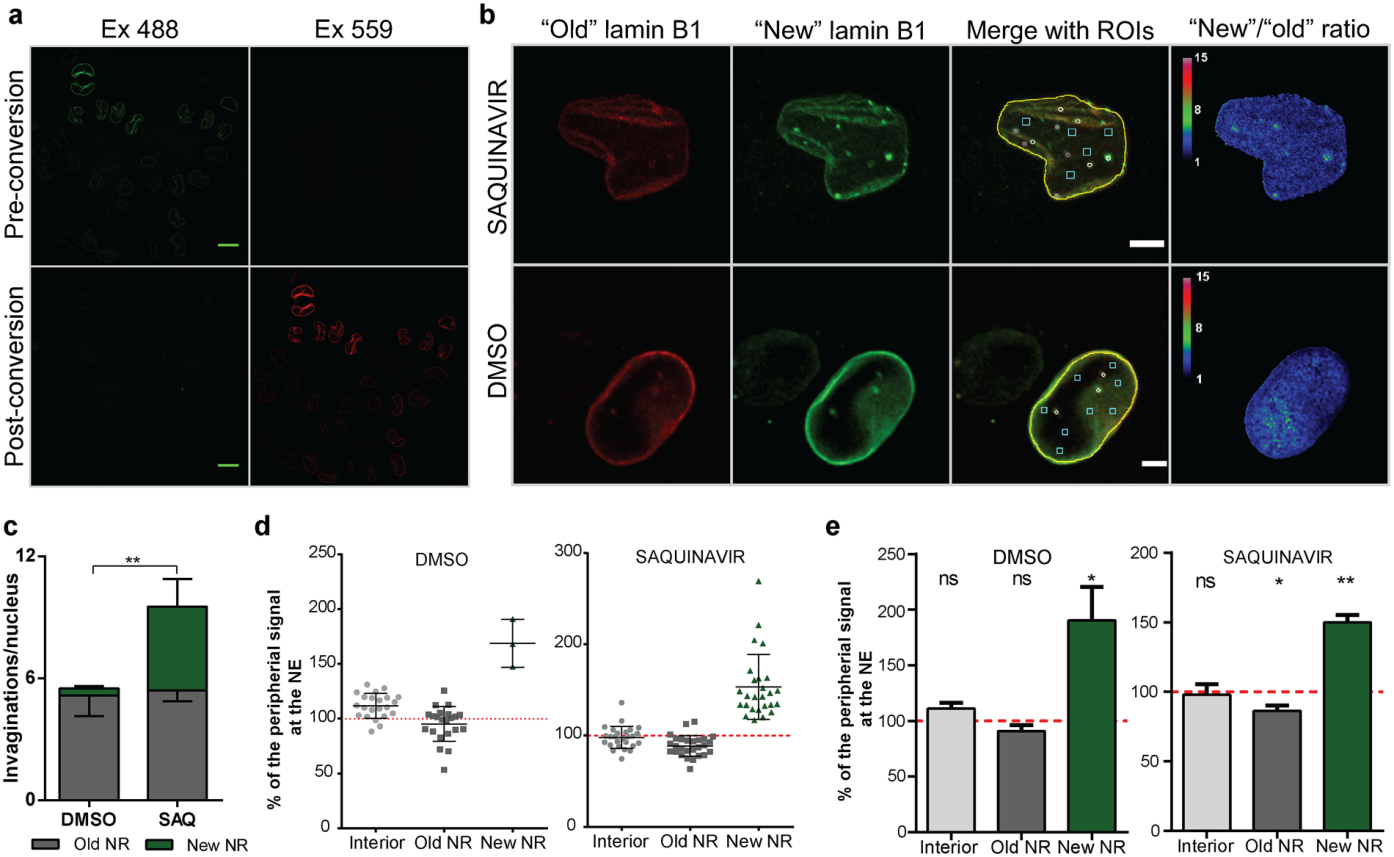
MAPLE3 photoconvertible tag allows tracking of nascent lamin B1 delivery to newly forming NR. (**a**) Confocal microscopy on HeLa cells 24 hours post-transfection with MAPLE3-lamin B1 encoding plasmid. Top panel corresponds to cells imaged before photoconversion (Pre-conversion) that emit green fluorescence upon excitation with 488 nm laser (Ex 488), but no red fluorescence upon excitation with 559 nm laser (Ex 559). Bottom panel (Post-conversion) presents the same cells imaged immediately after photo-conversion with 405 nm light, revealing complete switch in fluorescence emission from green (Ex 488) to red channel (Ex 559). Scale bar, 10 µm. (**b**) HeLa cells treated with either saquinavir or vehicle control (DMSO) and imaged 22–26 hours after complete photoconversion of MAPLE3-lamin B1. “Old” lamin B1 channel corresponds to the pool of lamin B1 existing in the cell immediately after photoconversion, while “new” lamin B1 corresponds to nascent copies of lamin B1 delivered to the nucleus post-conversion. Merge image shows Regions of Interest (ROIs) as follows: yellow line, nuclear periphery; white circles, old NR (structures visible in red channel); magenta circles, new NR (structures visible in green channel only); cyan squares, nuclear interior without structures. Ratiometric image comparing pixel intensities of green channel (“new” lamin B1) over red channel (“old” lamin B1). Scale bar, 5 µm. (**c**) NR invagination count in control (DMSO) and saquinavir-induced (SAQ) cells. Old NR has been identified in red channel, while new NR was defined by structures present in green channel only. Results from three independent experiments, 54 cells in total per condition; mean ± SD. (**d**) An example data plot from a single experiment showing distribution of “new”/”old” lamin B1 ratio at different nuclear structures and normalised to the nuclear rim ratio. (**e**) Pixel intensities of the ROIs defined in (B) based on the ratiometric images and normalised to the signal at the nuclear rim showing increased incorporation of nascent lamin B1 at the newly forming NR channels; results from three independent experiments, 54 cells in total; mean ± SD; **p < 0.001; *p < 0.05; ns, p not significant.

Since in this model NR formation relies on pathological accumulation of unprocessed prelamin A, we decided to study behaviour of other nuclear lamina components, such as lamin B1, post-translational processing of which is not directly affected by the drug treatment. To investigate whether nascent lamin B1 is selectively incorporated during NR formation, a cDNA encoding lamin B1 tagged with photoconvertible MAPLE3^15^ was expressed in HeLa cells. Unconverted MAPLE3-tagged lamin B1 can be visualised by confocal microscopy in the green channel. The tagged protein can then be fully photoconverted by exposure to 405 nm monochromatic light into a red fluorescent protein (Fig. 3a). After photoconversion, the appearance and distribution of newly synthesised MAPLE3-tagged lamin B1 (“new” lamin B1) over time can again be observed by confocal microscopy in green channel, while the pool of previously photoconverted MAPLE3-lamin B1 (the “old”) will remain red. We utilised this tool to investigate delivery of nascent copies of lamin B1 into the NE upon NR induction. After photoconversion, cells were allowed 18 hours to express new copies of MAPLE3-lamin B and then 4–8 hours of NR induction with saquinavir followed. Simultaneous confocal microscopy imaging in red and green channels can determine the ratio of nascent protein (“new” copies synthesised during particular time post photoconversion in green) relative to protein already present in a cell prior to photoconversion (“old” copies in red) (Fig. 3b). Regions of interest (ROIs) were drawn around nuclear structures as shown in Fig. 3b. White circles correspond to NR channels existing in the cell before photoconversion (hence visible in red channel); magenta circles correspond to newly formed NR (visible only in the green channel); while cyan squares were randomly drawn in the nuclear interior without any structures. Pixel intensities from ratiometric images were analysed in the ROIs and further normalised to the nuclear rim intensities in that cell. Such analysis revealed that upon saquinavir treatment the majority of cells form new NR, almost doubling the number of NR channels per nucleus (Fig. 3c). Furthermore, newly formed NR is significantly enriched in nascent lamin B1, while the NR tubules existing in cells prior to photoconversion show either no such trend or an inverted one (Fig. 3d,e). Since these are ratiometric measurements normalised to the NE ratios, this data confirms that the level of turnover of lamin B1 in the bulk NE lamina and the pre-existing NR lamina is measured to be identical. Moreover, since all NR profiles are counted as either old or new, and the red/green ratios for the two categories do not overlap (Fig. 3d), this confirms that newly-formed NR defined in this way contains an excess of newly made lamin B1.

Interestingly, such an approach can also identify newly formed NR in cells without drug induction. A limited number of cells (~10%) in DMSO control also developed new NR tubules during the course of the experiment (Fig. 3d,e), and these were also significantly enriched in nascent lamin B1 (Supplementary Fig. 4). While the rationale behind using the drug is to increase frequency of NR channels and to facilitate capturing the NR formation process as it occurs, the detection of newly formed NR channels in control cells not exposed to saquinavir present a more physiologically relevant situation. These events are less frequent than new NR formed as a result of pathological prelamin A accumulation, but experiments using photoconvertible tag on Lamin B1 confirm a statistically significant propensity for new physiological spontaneous NR channels to exhibit selective inclusion of nascent lamin B1. Thus, this could suggest that independent of the cause or regulation, mechanistically formation of both pathological and physiological NR exhibit overlapping properties.

Taken together, these studies, representing a combination of microscopy approaches to study nascent lamina precursors, strongly suggest that both experimentally induced and physiological NR formation occurs by local addition of new protein building blocks, rather than by rearrangement of a pre-existing lamina structure.

### NanoSIMS analysis demonstrates incorporation of nascent phospholipids into forming NR channels

Aside from the nuclear lamina component, the NR is, by definition, composed of at least one phospholipid bilayer nuclear membrane from which it originates. Moreover, it has been shown that knock-down of choline-phosphate cytidylyltransferase A (CCTα), the rate limiting enzyme for *de novo* synthesis of phosphatidylcholine (PC; the most abundant phospholipid in animal cell membranes) prevents formation of NR^14, 23^. Therefore, we next sought to determine whether nascent phospholipids are randomly delivered to the nuclear envelope by lateral diffusion from remote sites of synthesis and insertion, so enough membranous material is available for NR proliferation, or are synthesised and preferentially incorporated at sites of nascent NR formation. In order to follow nascent phospholipids, cells were pulsed with uniformly deuterated precursors, either choline or stearic acid. These deuterated precursors are biosynthetically incorporated into phospholipids, enabling pulse labelling of a nascent population synthesised at a particular time. The heavy atom tagged phospholipids were detected using the technique of Nanoscale Secondary Ion Mass Spectrometry (NanoSIMS)^16^. In this method a raster scanned ion beam is used to ablate a surface layer of the sectioned specimen to generate atomic fragments for high-resolution mass analysis. Correlation of 2D NanoSIMS maps with previous backscattered electron (BSE) imaging of the same region of the section allows in turn for analysis of the deuterated phospholipid distribution at specific cellular structures. Of note, due to the sample ablation required for NanoSIMS analysis; there will always be at least some mismatch between the surface BSE signal and a signal derived from the cumulative removal of sample to generate the NanoSIMS image.

**Figure 4:**
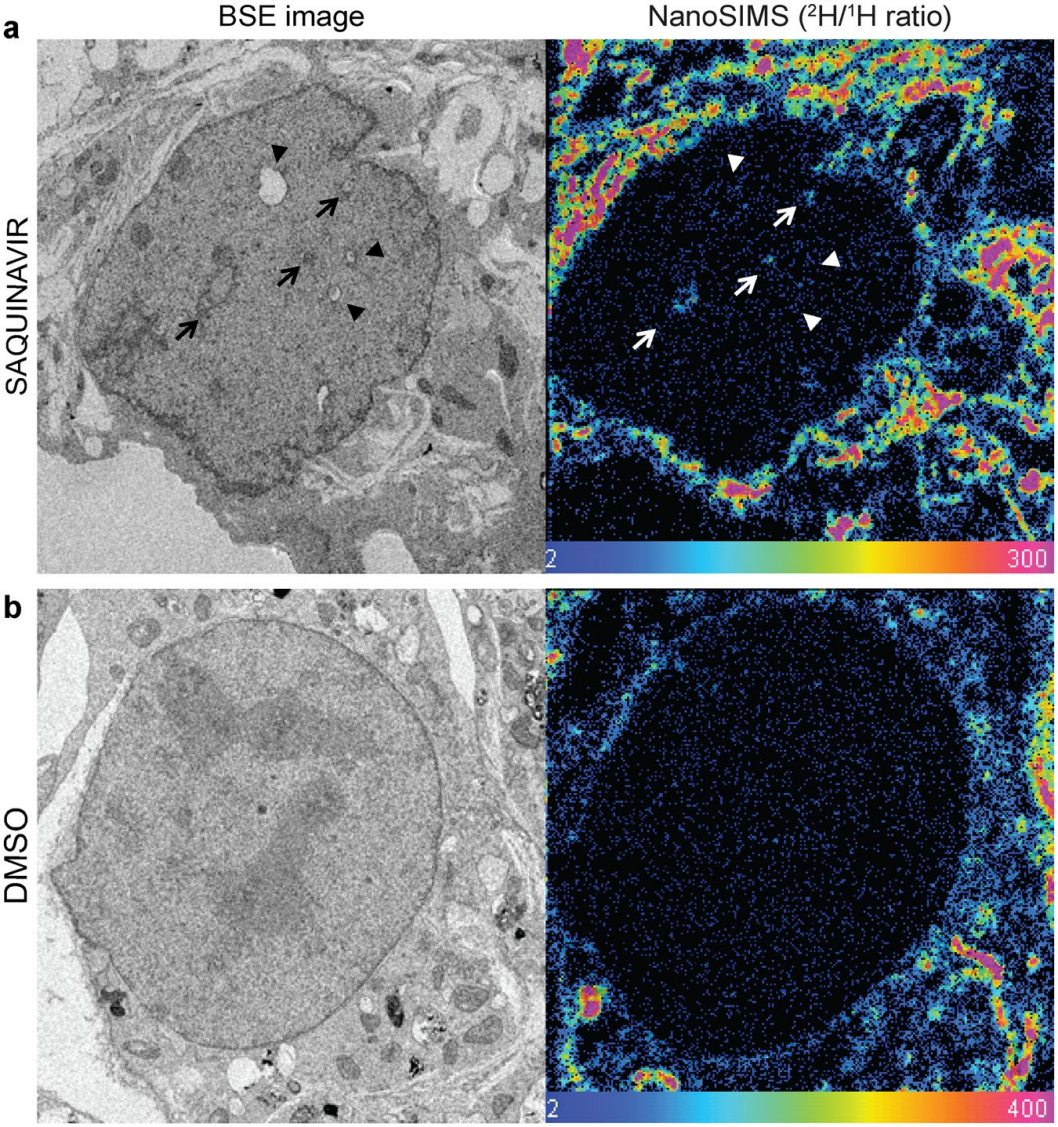
Detection of nascent phospholipids during NR formation by pulse labelling with deuterated stearate. (**a**) Representative backscattered electron (BSE) image of a saquinavir-treated mouse preadipocyte with black arrows indicating NR tubules with a corresponding image from NanoSIMS showing enrichment of deuterium signal (^2^H/^1^H ratio) indicating nascent fatty acyl species, also at the NR foci (indicated with white arrows); black arrowheads in BSE image correspond to NR presumably present in the nucleus prior to ^2^H pulse, hence not showing enrichment in deuterium signal in the NanoSIMS image (white arroheads). (**b**) BSE and NanoSIMS images of a control cell treated with DMSO vehicle showing lack of NR tubules and no deuterium enrichment within the nucleus, and undistorted nuclear boundary. Colour scale of NanoSIMS images 2–400 equals 0.02–4% of ^2^H/^1^H.

Mouse preadipocytes were selected as a model cell line in this study due to their efficient uptake of fatty acids from culture medium^24^. Furthermore, similar to other cell lines, they respond to saquinavir by prelamin A accumulation and, subsequently, formation of new NR channels (Supplementary Fig. 5). Initially, cells were pulse labelled with uniformly deuterated stearate, which can serve as a source of fatty acyl moieties. As revealed by BSE imaging, mouse preadipocytes treated with saquinavir exhibited distorted nuclear periphery with membranous NR structures within the nucleus as previously described (Fig. 4a), whereas DMSO-treated control cells retained a smooth and uniform nuclear membrane (Fig. 4b). This is consistent with our results on other cell types. When these images were correlated with the ^2^H/^1^H ratio maps obtained by NanoSIMS analysis, an increased ^2^H/^1^H ratio was seen at the endoplasmic reticulum (ER) in both conditions, indicating that cells efficiently absorbed deuterated stearate from the growth medium. Furthermore, this is in agreement with the role of the ER as one of the main sites, other than the Golgi apparatus, of *de novo* phospholipid biosynthesis^25^. The bulk nuclear boundary in control cells displayed very modest ^2^H enrichment indicating that these membranes were stable and not rapidly turning over during the experiment. Conversely, saquinavir treated cells demonstrated a much higher ^2^H signal around the nuclear envelope when compared to control cells, suggesting dynamic membrane rearrangements requiring the incorporation of nascent phospholipids. Furthermore, intranuclear channels (indicated by arrows in Fig. 4) observed in nuclei from these cells were also enriched in deuterium, therefore showing incorporation of newly synthesised phospholipids into forming NR. As seen in the BSE image, however, NR channels without elevated ratios in the corresponding NanoSIMS image are also present (indicated by arrowheads). Presumably they represent pre-existing NR which has not recruited new (deuterated) membrane components during the pulse-chase period. This is consistent with the observation from the photo-convertible lamin B1 experiments that it is not NR *per se* that has high incorporation ratios, but newly formed NR.

### Phosphatidylcholine synthesised *de novo* is incorporated into forming NR

**Figure 5:**
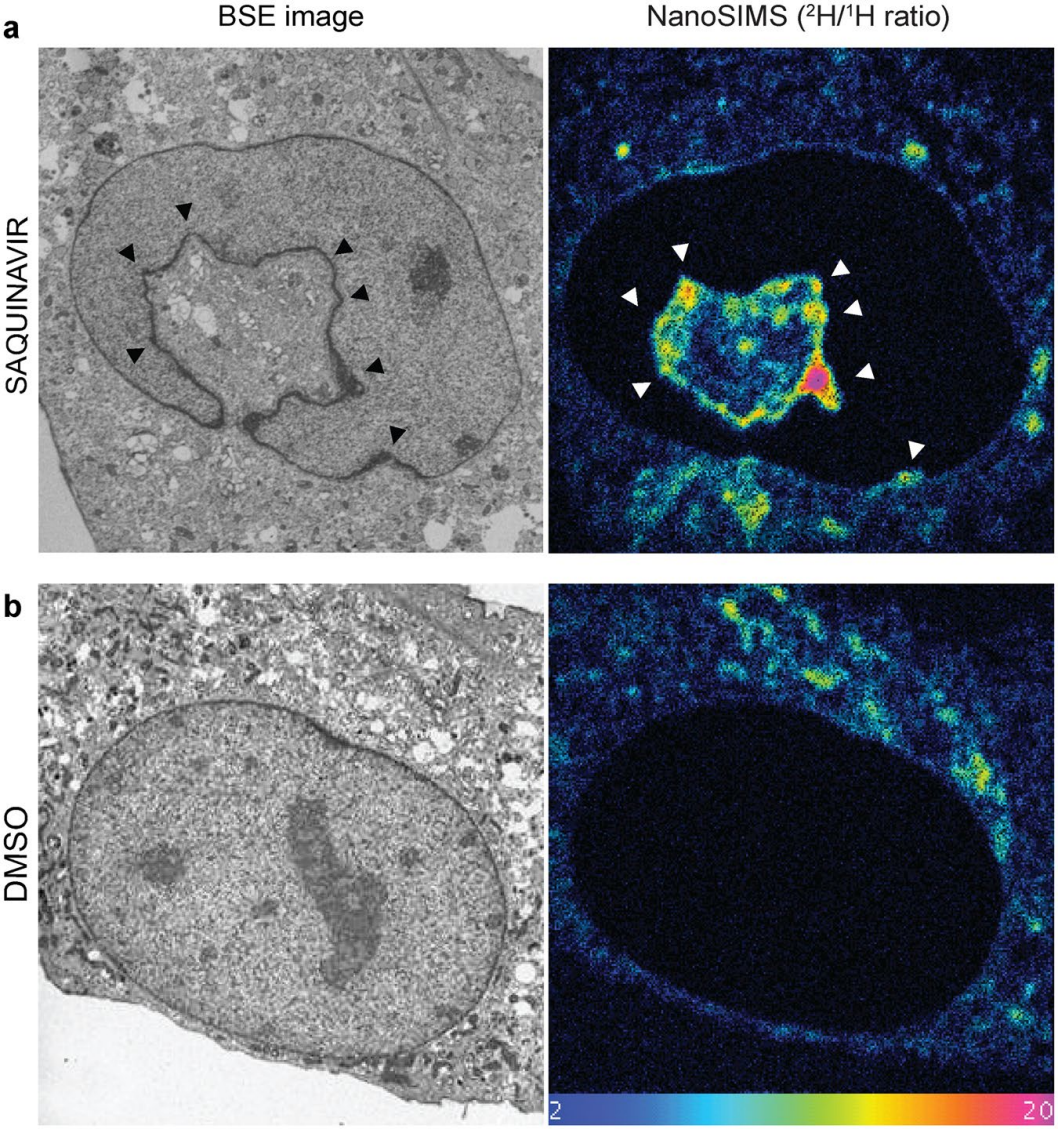
Detection of nascent phospholipids by pulse labelling with deuterated choline during NR formation. (**a**) Representative backscattered electron (BSE) image of a saquinavir-treated mouse preadipocyte (black arrowheads point towards high membrane curvature indicating NR initiation sites) with a corresponding image from NanoSIMS showing enrichment of deuterium signal (^2^H/^1^H ratio) revealing nascent phospholipids incorporated directly into forming NR (white arrowheads). (**b**) BSE and NanoSIMS images of a control cell treated with DMSO vehicle showing lack of NR tubules and no deuterium enrichment within the nucleus, and undistorted nuclear boundary. Colour scale of NanoSIMS images 2–20 equals 0.02–0.2% of ^2^H/^1^H ratio.

Synthesis of phospholipids, apart from fatty acid tails, also requires head-group moieties, choline in the case of PC, as a substrate. Since stearate enters a wider range of metabolic pathways than choline does, using deuterated choline chloride allows for even more specific labelling of nascent phosphatidylcholine, the most abundant phospholipid in a cell. Therefore, in parallel experiments, mouse preadipocytes were pulse labelled with deuterated choline chloride in the presence of saquinavir. Following analysis by the NanoSIMS, behaviour similar to pulse-labelling with deuterated stearate was observed. Upon saquinavir treatment, the nuclear membrane became much more dynamic with focal points of nascent phospholipid incorporation. The highest abundance of nascent phospholipids was revealed though, at the sites of increased membrane curvature on intranuclear structures corresponding to NR formation sites (Fig. 5 and Supplementary Movie 1). This not only demonstrates the applicability of our NanoSIMS approach in following nascent phospholipid pool in a cell, but also further supports the hypothesis that newly forming NR requires machinery synthesising *de novo* building blocks for NR expansion.

### 3D NanoSIMS shows spatial distribution of nascent phospholipids in a nucleus

**Figure 6:**
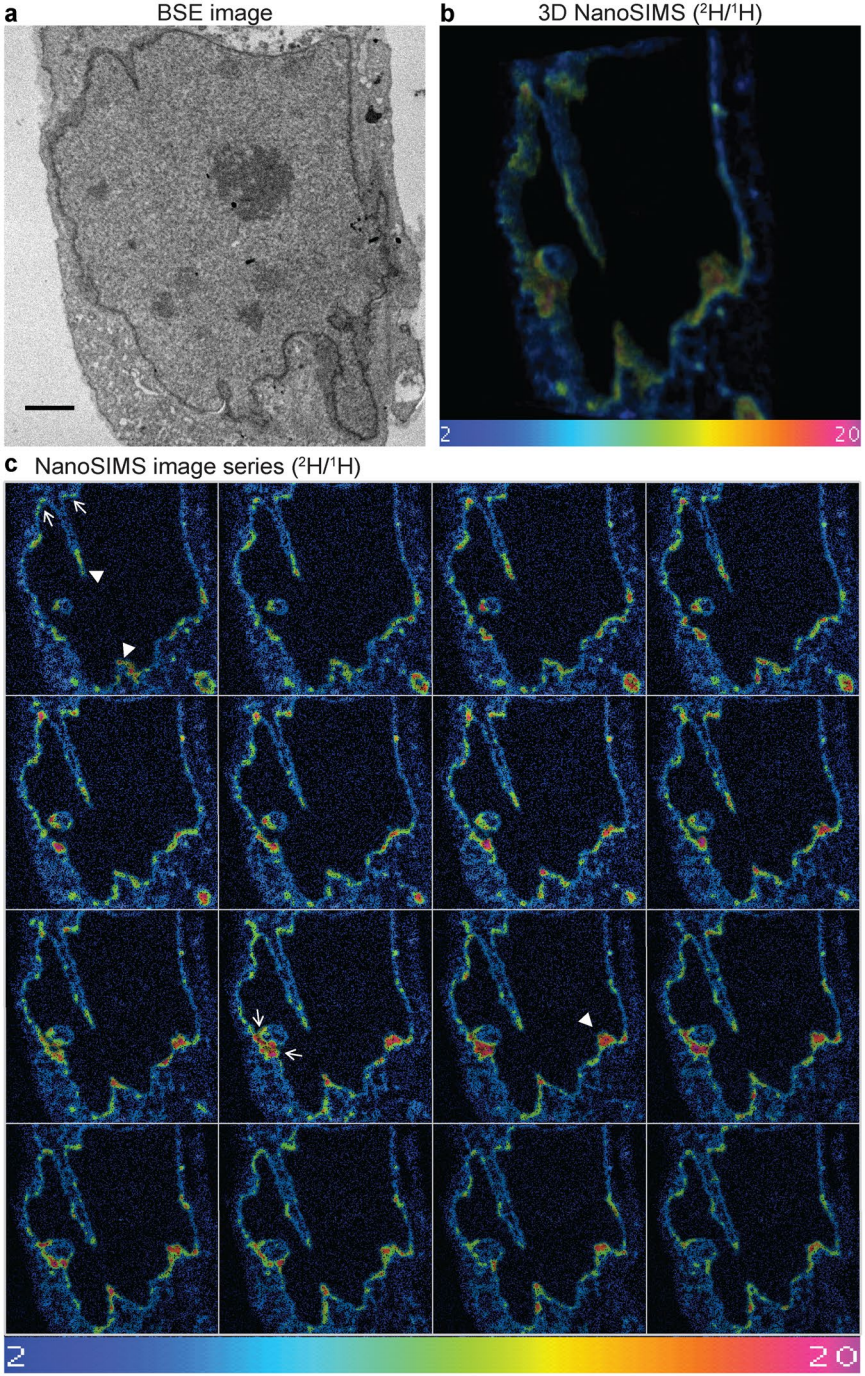
3D NanoSIMS of nascent phospholipid distribution during NR formation. (**a**) Representative backscattered electron (BSE) image of a saquinavir-treated mouse preadipocyte pulsed labelled with deuterated choline; scale bar 2 µm. (**b**) 3D reconstruction of NanoSIMS analysis of subsequent layers of the specimen shown in (**a**). (**c**) Panel of NanoSIMS images used for the 3D reconstruction shown in (**b**); examples of tip and base region of NR tubules are indicated by arrowheads and arrows, correspondingly; a single image corresponds to ~10 nm specimen thickness. Colour scale of NanoSIMS images 2–20 equals 0.02–0.2% of ^2^H/^1^H ratio. See corresponding Supplementary Movies 2 and 3.

**Figure 7:**
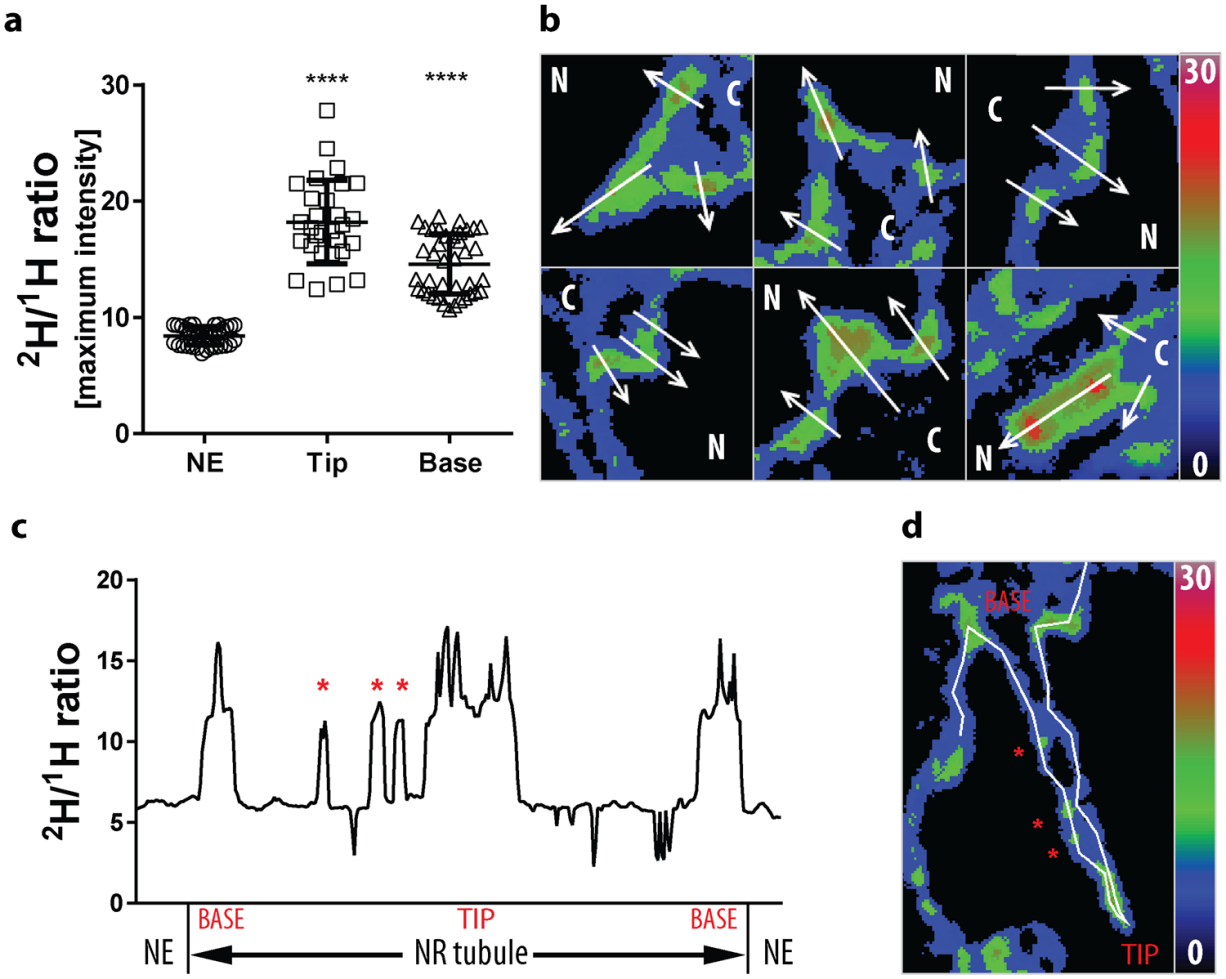
Quantification of the nascent phospholipid enrichment at NR structures in the NanoSIMS experiments. (**a**) Scatter plot showing maximum intensity of ^2^H/^1^H ratio at the bulk nuclear envelope (NE), and the tip and base regions of NR invaginations in mouse preadipocytes pulse labelled with deuterated choline chloride; mean ± SD indicated; ****p < 0.0001. (**b**) Examples of ratiometric images used for quantification of the ^2^H/^1^H ratios with indicated line profiles corresponding to the base region (outer arrows) or tip (middle arrow) of NR tubules; N, nucleoplasm; C, cytoplasm. Note the different appearance of these images from NanoSIMS images presented in other figures, due to using ratiometric images for measurements rather than HSI images. (**c**) Plot profile of ^2^H/^1^H ratio along a typical invagination shown in (**d**); asterisks correspond to focal PC inclusion sites along the tubule length. Colour scale of 0–30 equals 0–0.3% of ^2^H/^1^H ratio.

Next, the nascent phospholipid distribution along the NR structures was further analysed by devising 3D NanoSIMS. This investigation was performed by the step-wise ablation of layers from the specimen surface with the ion beam, followed by analysis of the atomic mass spectra from each consecutive tier by the NanoSIMS. This approach allowed for the three dimensional imaging of nascent phospholipids during the process of NR formation and further highlighted that growing NR channels require *de novo* synthesised phospholipids (Fig. 6, Supplementary Movies 1–5, and Supplementary Fig. 6 and 7). Moreover, our data also revealed a differential incorporation rate of nascent phospholipids even along the NR channels (Fig. 7). The base and the tip of the forming NR tubes appear to be the main inclusion sites showing the highest ^2^H/^1^H ratio, but also unevenly distributed focal hot spots of PC incorporation were detected along NR channels. This observation is further supported by the 3D NanoSIMS analysis of cells pulse-chase labelled with deuterated stearate (Supplementary Fig. 8).

## Discussion

The NR appears to be a dynamic structure with many pathways contributing to its formation. The data presented here demonstrate that NR formation requires the incorporation of new components, both protein (such as lamins) and nascent membrane phospholipids, rather than rearrangement of the pre-existing NE. Nuclear architecture and morphology is complex, dynamic and affected by various forces both from within the nucleus and/or from the cytoplasm^26–29^. The functional implications of such changes are not well understood. The intriguing question is what are the forces behind NR formation that introduce such extensive alterations to the NE? Several scenarios are possible regarding the models of NR formation^30^. Nuclear architecture may be defined by interactions between chromatin and the NE^31, 32^; indeed, abundant focal interaction sites called LADs (Lamina-associated domains) linking nuclear lamina and underlying chromatin have been identified by methylase proximity labelling^33^. Furthermore, in an interphase nucleus, dynamic chromatin movements occur as a result of chromosome condensation^34, 35^. Thus, NR invaginations could be driven by rearrangements of chromatin tethered to the NE and pulling in the nuclear membrane. This observation was made for NR formation in polytene nuclei from Drosophila melanogaster salivary glands^36^. In an alternative scenario, the pressure may come from outside of the nucleus. It is well established that the cytoskeleton can counterbalance internal forces acting on chromatin and the nuclear lamina, thus playing a pivotal role in stabilisation of nuclear architecture^27, 28, 37^. It is possible then that the cytoskeleton exerts forces on the NE and pushes it in. In fact, it has been shown that type II NR invaginations contain microtubules and microfilaments in their core^2, 3, 38, 39^. The third scenario could suggest the existence of a dedicated machinery that assembles the NR structure *de novo*, rather than through rearrangement of elements already incorporated into the NE. Indeed, multiple mechanisms exist in a cell that are responsible for a focal and localised induction of membrane curvature and invaginations of plasma membrane and other organelles^40^, such as clathrin-mediated endocytosis^41, 42^, endophilin-mediated endocytosis^43^, caveolae formation^44, 45^, and reticulon-dependent tubular ER formation^46^ to name just a few. These orchestrated processes require regulated interaction of mediator proteins and can result in changes of membrane morphologies, leading to formation of tubular channels and vesicles.

Our data supports the *de novo* assembly model, and shows that formation of the NR requires incorporation of new components directly into proliferating NR tubules. It should be noted though that this does not preclude the other two models, and that they may co-exist. Whatever the physical strain on the NE, however, there is a need for machinery that will assemble the NR structures *de novo*. Super resolution microscopy of fibroblasts exposed to saquinavir revealed that prelamin A is enriched at the NR structures. This is the newly synthesised protein; hence, it strongly suggests that forming NR displays a need for building blocks that are delivered directly to the proliferating NR channels. Since this is a pathological model of forced prelamin A accumulation, it is important to consider whether the same process occurs during physiological NR formation. This cannot be tested by following prelamin A accumulation, so another approach is needed. The use of a photoconvertible tag on a reporter protein as described here provides a generalised method for following the distribution of nascent protein incorporation. This is a powerful method that can be applied at steady state as well as after experimental manipulations of cell state. The experiments with photoconvertible MAPLE3-lamin B1 demonstrate the same local selective incorporation as seen during forced prelamin A accumulation, but extend this observation by revealing that spontaneously forming NR in untreated cells also form by a process involving selective targeting of nascent lamin proteins.

The proliferation of NR requires additional membrane area to be incorporated into the NE. This could arise by diffusional flow from connected stores such as ER membrane, or be provided by local synthesis of new membrane components. In order to distinguish between these possibilities it is necessary to image the subcellular distribution of nascent phospholipids, a demanding challenge requiring a pulse-chase strategy for labelling this subset of molecules^47^. Here we use stable isotope pulse-chase experiments analysed by NanoSIMS to confirm that the added membrane of forming NR is highly enriched with newly synthesised phospholipid molecules. It has been previously demonstrated that NR formation depends on CCTα, the rate limiting enzyme in membrane phospholipid biosynthesis^14, 23^. Indeed, while reduction of CCTα expression selectively prevents NR formation, other consequences of prelamin A accumulation less dependent on increased membrane area (e.g. a more convoluted NE perimeter) remain intact^14^. The origin of the membrane from which NR was constituted during its proliferation was not examined in that study. Here we show that newly synthesised phospholipids are delivered directly to the sites of NR proliferation, with the highest rate of their incorporation at the base and tip of the forming NR channel. In addition, small foci with higher lipid incorporation rates were also observed along the channel length. This is consistent with observations that CCTα is also unevenly distributed along the NR tubules^14, 48^, suggesting that lipid synthetic machinery may be focal. Although CCTα mediates CDP-choline synthesis, the rate-limiting step in PC production, this enzyme does not make PC itself. It is the role of either the cholinephosphotransferase (CPT) or the choline/ethanolaminephosphotransferase (CEPT)^49^. Interestingly, the CEPT was found to localise to NE^50^, and when overexpressed it was also present at NR structures along with CCTα^48^. Thus, such observations could explain how CDP-choline produced by CCTα is channelled to CEPT located at nuclear membranes, and it agrees with the proposed focal PC biosynthesis at nuclear invaginations.

Moreover, smaller foci along the NR tubule could mark points of NR expansion and perhaps facilitate sites of further invaginations when the NR forms branched structures. Such a side branch of NR emerging from a site of high ^2^H/^1^H ratio can be seen in Figs 5–7 and Supplementary Movies 2–5. A striking feature of the ^2^H/^1^H ratio maps is the sharp boundary of the zones of nascent phospholipid enrichment; the deuterated product accumulates over a pulse interval of six (stearate) or twelve (choline) hours, and might be expected to diffuse widely over this period. The absence of such diffusion implies a barrier to free movement of newly incorporated phospholipids at the sites of maximum curvature (the base and tip of invaginating tubes, and the base of lateral branches). These could potentially be attributed to recruitment and retention of proteins aiding in maintaining membrane curvature. For example, mammalian reticulons and DP1 can form membrane embedded oligomers that remain relatively immobile^51, 52^, and hence similar mechanism could be present at forming NR. Such proteins, while aiding in membrane tubulation, possibly could also be the barrier to rapid phospholipid diffusion.

The NR, despite being a widespread feature of nuclear structure, remains underappreciated. Nonetheless, the results described here shows that the ability of a cell to form such a complex structure during interphase can now be ascribed at least in part to a localised machinery that incorporates nascent building blocks. Furthermore, the NanoSIMS approach presented here can aid in studying of the dynamics of cellular membranes in general, while MAPLE3 photoconvertible tagging can be applicable to investigate also other cellular processes.

## Methods

### Mammalian cell culture, drug treatment and transfection

Human dermal fibroblast (HDF) primary cells^53^ from healthy volunteers were kindly provided by Dr Winnok De Vos, Department of Molecular Cell Biology, CARIM-School for Cardiovascular Diseases, Maastricht University, The Netherlands. HeLa cells were obtained from laboratory stocks. Mouse preadipocytes (3T3-F442A) were supplied by the Public Health Protection England from the European Collection of Authenticated Cell Cultures (ECACC). Cells, unless otherwise stated, were cultured in Dulbecco’s Modified Eagle Medium (DMEM) supplemented with 10% foetal calf serum (FCS), 1% non-essential amino acids, and penicillin and streptomycin antibiotics (100 units/ml). Both saquinavir and darunavir drugs were added to CO_2_- and temperature-equilibrated cell culture medium to 20 µM concentration. Daily growth medium change with the addition of fresh drug followed unless otherwise stated. Negative controls were incubated with the appropriate volume of the DMSO vehicle alone. Plasmid and siRNA transfections were performed with Lipofectamine 2000 (Invitrogen, Carlsbad, California) or Lipofectamine RNAiMAX (Invitrogen) respectively, following manufacturer’s recommendations. Zmpste24 and control siRNA SmartPools were purchased from Dharmacon.

### Western blot analysis

Equal numbers of cells were resuspended in sample lysis buffer (Thermo Scientific, Waltham, Massachusetts) supplemented with 10 mM DTT, followed by a five minute incubation at 90 °C. Cell lysates were run in precast 4–12% NuPAGE Bis-Tris gels (Novex, Carlsbad, California) in MOPS buffer (50 mM MOPS, 50 mM Tris Base, 0.1% SDS, 1 mM EDTA, pH 7.7) (Novex) with the addition of NuPAGE Antioxidant (Novex) at a constant voltage of 200 V for one hour. Proteins from the gels were transferred to nitrocellulose membrane using the Mini Trans-Blot Electrophoretic Transfer (Bio-Rad, Hercules, California), and then immunolabelling of the membrane followed. First, the membrane was blocked in 5% non-fat dried milk in PBST (PBS, 0.05% Tween) for 30 minutes at room temperature on a rocker. Immunolabelling with primary and secondary antibodies was performed in the blocking solution with rocking. Primary antibodies used were mouse anti – lamin A/C (4C11, Active Motif, Carlsbad, California) and rabbit anti – β actin (AC-15, Abcam, Cambridge, UK), while secondary antibodies were peroxidase conjugated goat anti – mouse IgG (A4416, Sigma, St. Louis, Missouri) or anti – rabbit IgG (A6154, Sigma). Incubation with primary antibody was done either for one hour at room temperature, or overnight at 4 °C, while the secondary antibody was left for one hour at room temperature. Each of the antibody incubations was followed by 3 five-minute washes with PBST buffer. The membrane was then developed with the Immobilon™ Western Chemiluminescent HRP Substrate (Merck Millipore, Billerica, Massachusetts); the signal was detected by exposure to an x-ray film and developed on a Kodak X-OMAT 2000 processor.

### Immunofluorescent labelling and confocal microscopy

Cells were seeded on glass coverslips and grown as a monolayer. They were washed in PBS prior to fixation with 4% paraformaldehyde (Electron Microscopy Sciences, Hatfield, Pennsylvania) in PBS for ten minutes at room temperature. Free aldehyde groups were subsequently quenched with 25 mM glycine in PBS for five minutes. Cell permeabilisation in 0.5% Triton X-100 in PBS followed for five minutes. Cells were blocked in 0.5% fish skin gelatin in PBS for either 30 minutes at room temperature or overnight at 4 °C. Antibodies were diluted in this blocking solution. Immunolabelling with primary and secondary antibodies followed, each for one hour at room temperature, with three PBS washes after each antibody. Primary antibodies used were mouse anti – lamin B1 (8D1^54^), and anti – prelamin A (C-20; Santa Cruz, Dallas, Texas). Secondary antibodies used were donkey anti-mouse and donkey anti – goat conjugated to Alexa Fluor 488 and Alexa Fluor 647 (Invitrogen), respectively. Coverslips with immunolabelled cell monolayer were then dipped in ultrapure water and immediately mounted on glass microscopy slides with Mowiol mounting solution supplemented with DAPI (0.2 µg/ml). Slides were left at 4 °C in the dark overnight to allow the Mowiol to set.

Immunostained cells were imaged on the LSM5 Zeiss Inverted 510 META laser scanning microscope (Zeiss, Oberkochen, Germany) using a Plan Apo 63 × 1.4 NA oil immersion lens. Images were acquired using Zen2009 operating software. Collected images were analysed in ImageJ v1.45 s (Wayne Rasband, NIH, USA).

### Structured illumination microscopy with 3D reconstruction (3D-SIM)

Primary human dermal fibroblasts (HDF) were seeded on high precision coverslips (22 × 22 mm), thickness no. 1.5 H (170 µm ± 5 µm) (Marienfeld, Lauda-Königshofen, Germany) in 6-well plates and returned to the incubator to allow time to adhere and spread. 20 µM saquinavir treatment followed for 48 hours. At the time of fixation, cells were reaching 80% confluency. HDFs were washed twice with 2 ml of PBS for five minutes each time and fixed in 4% paraformaldehyde (Electron Microscopy Sciences) in PBS for ten minutes. Fixative was aspirated and 2 ml of 25 mM glycine solution in PBS per well were added for five minutes to quench free aldehyde groups. Cells were permeabilised by replacing the glycine solution with 2 ml of 0.5% Triton X-100 in PBS and incubating for five minutes before blocking with 2 ml of 0.5% FSG in PBS for 30 min at room temperature or overnight at 4 °C. Primary and secondary antibodies were diluted in blocking solution and incubation for each was one hour, followed by 3 five-minute washes with PBST (PBS 0.05% Tween). Primary antibodies used were: goat anti-lamin B (C-20; Santa Cruz) mouse anti – lamin A/C (4C11; Active Motif), and goat anti – prelamin A (C-20; Santa Cruz). Secondary antibodies used were donkey anti-mouse and donkey anti – goat conjugated to Alexa Fluor 594 and Alexa Fluor 488 (Invitrogen), respectively. DNA was labelled with 1 ml of 2 µg/ml DAPI in PBS for five minutes before transferring the coverslips to deionized water for five minutes. Coverslips were mounted in 13 µl of Vectashield H-1000 (Vector Laboratories, Burlingame, California) and sealed with nail polish immediately.

Slides were imaged on OMX V3 Blaze microscope (GE Healthcare, Little Chalfont, UK) equipped with a 60x/1.42 oil UPlanSApo objective (Olympus, Tokyo, Japan), 405 nm, 488 nm, 593 nm diode lasers, and sCMOS cameras (PCO, Kelheim, Germany). The instrument permitted acquisition of 3D-SIM image stacks with five phases, three angles per image plane, and 0.125 μm z-distance between sections. To minimize spherical aberrations, immersion oil of pre-selected refractive indices (RIs) was selected to match the respective optical transfer functions (OTFs) used. For imaging thick specimens, best results were typically obtained with RI 1.514 for depth adjustment in the region of optimal reconstruction a few μm into the sample. The raw data was computationally reconstructed with SoftWoRx 6.0 (Applied Precision, Issaquah, Washington). Wiener filter settings 0.002 and channel specifically measured OTFs were applied to generate a super-resolution 3D image stack, obtaining lateral (x-y) resolution of up to 100–130 nm and an axial (z) resolution of ∼300 nm. Images from the different colour channels were registered with the alignment parameter obtained from calibration measurements with 0.2 μm diameter TetraSpeck beads (Life Technologies, Carlsbad, California) using the OMX Editor software. Reconstructed images were analysed in Volocity and Imaris software, while prelamin A brightness at different nuclear loci was quantified in ImageJ.

### Confocal microscopy on cells expressing photoconvertible MAPLE3-lamin B1

Initially, lamin B1 cDNA was cloned into mKikGR-C1 (a gift from Michael Davidson; Addgene plasmid # 54656) between BglII and XmaI restriction sites. Then region encoding mKikGR tag was excised using NheI and BglII restriction enzymes (New England Biolab, Ipswich, Massachusetts), and photoconvertible MAPLE3 tag^15^ was inserted instead.

HeLa cells were transfected with MAPLE3-lamin B1 construct and subjected to live cell imaging on Olympus FV1200 laser scanning microscope equipped with temperature and CO_2_ chamber for live cell work. 60 × 1.4 NA oil objective was used and images were acquired with Fluoview software (Olympus). 24 hours post transfection cells were scanned using 488 nm and 559 nm laser wavelengths to confirm emission of fluorescence in green channel but not red. Complete photoconversion of green fluorescence to red was achieved by exposing cells to UV illumination source from U-HGLGPS 100 W mercury lamp for 60 seconds at 12% lamp power, and confirmed by immediate cell imaging with 488 nm and 559 nm laser wavelengths. After photoconversion, cells were allowed 18 hours to express new copies of MAPLE3-lamin B1 and then 4–8 hours of NR induction with 20 µM saquinavir followed. Z-stack images of 0.44 µm interval were acquired by scanning with 488 nm and 559 nm laser lines and were used for analysis of nascent lamin B1 (green channel) distribution in regard to lamin B1 pool existing in cells immediately after the photoconversion event (red channel). Corresponding phase images for assessment of cell morphology and fitness were also acquired to exclude dying/unhealthy cells from the analysis.

Image analysis was performed in ImageJ software. Median filter 3 × 3 was applied to all images, followed by Z-projection of maximum intensity. Cytoplasmic area was masked and only nuclear lamin B1 signal was used for calculation of ratiometric images. Regions of interest (ROIs) were defined based on fluorescent channel images and applied to ratiometric images. Pixel intensities at ROIs were exported to GraphPad Prism and statistical analysis followed.

### Correlative backscattered electron microscopy and NanoSIMS imaging

#### Preparation of deuterated substrates for phospholipid labelling

Sample preparation followed protocol described elsewhere^55^ with detailed procedure given below. Deuterated Choline Chloride D9 (CDN Isotopes, Quebec, Canada) was dissolved in culture medium to 1 M concentration and used at 80 mM for cell treatment.

One mM Stearic Acid D35 stock solution was prepared by dissolving 3.2 mg of Stearic Acid D35 (Sigma) in 2 ml of ethanol. It was then complexed with 400 µl of 100 mM solution of NaOH in ethanol. Alcohol was evaporated with nitrogen gas obtaining fatty acid soaps. They were dissolved in 0.5 ml of hot ultrapure water and kept in a 55 °C water bath for ten minutes. One gram of fatty acid-free bovine serum albumin (Sigma) was dissolved in 9.5 ml DMEM, warmed to 55 °C, added to the dissolved fatty acid soaps, and vortex mixed for ten seconds, followed by a further ten minute incubation at 55 °C. The 1 mM deuterated Stearic Acid D35 stock solution was sterilized by filtration, aliquoted and kept at −20 °C. Upon thawing, the stearic acid sample was warmed to 55 °C to dissolve the precipitate (if formed) and subsequently cooled to 37 °C before adding to cells.

#### Cell treatment

Mouse preadipocytes were seeded on 13 mm plastic coverslips (Thermanox; VWR, Radnor, Pennsylvania) in 24-well plates at 50% confluency and left overnight in the cell culture incubator. Labelling of fatty acyl moieties with deuterated stearate was performed the next day. Cells were treated with 20 µM saquinavir for 12 hours and with 10 µM Stearic Acid D35 for the last six hours of saquinavir treatment. In order to increase efficiency of Choline Chloride D9 uptake by cells, culture medium was changed to medium supplemented with 1% FCS for 24 h. Then 80 mM Choline Chloride D9 was added, together with 20 µM saquinavir for 12 hours.

#### Cell fixation and specimen preparation for imaging

Upon treatment completion, cells on coverslips were washed with PBS and fixed at room temperature for 20 minutes by adding 500 µl of fixative warmed to 37 °C. Solution of 4% paraformaldehyde and 1% glutyraldehyde in 100 mM PIPES pH 7.4 was used as the primary fixative. Secondary fixation in 2.5% glutyraldehyde in 100 mM PIPES pH 7.4 followed. The cells were incubated for one hour at room temperature, then transferred to 4 °C and left overnight.

The next day, samples were washed three times for ten minutes each with 100 mM PIPES pH 7.4, followed by osmication with 1% osmium tetroxide in 100 mM PIPES pH 7.4 for one hour and washed in deionized water for 20 minutes. The cells then went through a graded ethanol series, first at 50% ethanol for 15 minutes, then 70% ethanol overnight at 4 °C, then 90% ethanol for 15 minutes, then 95% ethanol for 15 minutes, and finally 100% ethanol for two hours with three solution changes during this time. Gradual infiltration with Agar 100 epoxy resin (Agar Scientific, Stansted, UK) followed, starting with 25% resin for one hour, then 50% resin for two hours, then 75% resin for one hour, and 100% resin overnight. The next day, samples were transferred twice to fresh 100% resin for three hours each time. The cells were resin-embedded by inverting the coverslips, cells facing down, onto an embedding capsule (BEEM, Electron Microscopy Sciences) filled with fresh resin and left for 24 hours at 60 °C for polymerization. Polymerized blocks were then submerged in liquid nitrogen and the coverslips were snapped off to leave the cells embedded as a monolayer in the resin. The specimen was cut to obtain semi-thin sections of 0.5–1 µm using a Leica UC7 ultramicrotome with a diamond knife (Diatome, Biel, Switzerland). Each section was floated on a droplet of water on a 15 nm platinum coated coverslip. These were placed on a 60 °C heating block and allowed to dry.

#### Backscattered electron microscopy imaging

Before starting backscattered electron (BSE) imaging, areas of interest were recorded by optical microscope. Sections were then transferred to the NVision FIB scanning electron microscope and BSE images were acquired with a 2 kV incident beam with a standard aperture (30 µm) and 5-mm working distance.

#### Nanoscale Secondary Ion Mass Spectrometry

Upon the completion of BSE imaging, the sections were coated with 5 nm of platinum in a Cressington 208HR high-resolution sputter coater (Cressington Scientific Instruments Ltd., Watford, UK) to provide the surface conductive for Nanoscale Secondary Ion Mass Spectrometry (NanoSIMS) imaging on Cameca NanoSIMS50 (Cameca, Gennevilliers, France).

First, the Cs^+^ primary beam was used to remove the platinum on the surface at selected locations, simultaneously implementing a Cs^+^ dose of 1.0 × 10^17^ ions/cm^2^. Small apertures (D1 = 3 or D1 = 4) were used for imaging a single cell in order to match the size of primary beam to the pixel size. The instrument was tuned for ^2^H^−^ and ^1^H^−^ to allow calculation of the ^2^H/^1^H ratio. The NanoSIMS images were acquired with a dwell time of 30,000 µs per pixel for 256 × 256 pixel images. A median filter with radius of 3 pixels was applied to the Hue Saturation Intensity (HSI) image. A single NanoSIMS image corresponds to ~10 nm specimen thickness.

#### Image analysis and processing

The BSE and NanoSIMS images were aligned, and the local ^2^H/^1^H ratio quantified in ImageJ software. ^2^H/^1^H ratio across images and experiments was normalised to the bulk signal at the NE with exclusion of enrichment areas. Data from ImageJ was then imported to Excel and GraphPad Prism for further analysis.

### Statistics

Unless indicated otherwise, results represent at least three independent biological replicates. Scatter plots show pooled data, while bar graphs represent the means with standard deviation of the results from independent repeats. Exact number of repeats and cells in a cohort is indicated in figure legends. A two-tailed, unpaired Student’s t-test was employed to determine statistical significance of the results.

## Electronic supplementary material

Supplementary Movie 1

Supplementary Movie 2

Supplementary Movie 3

Supplementary Movie 4

Supplementary Movie 5

Supplementary material
