# Supplementary material for: Formation of a nucleoplasmic reticulum requires de novo assembly of nascent phospholipids and shows preferential incorporation of nascent lamins
Source: Sci Rep. 2017 Aug 7;7:7454. doi: 10.1038/s41598-017-07614-w (PMC5547041; doi:10.1038/s41598-017-07614-w)
Supplement: Supplementary file 6 — Supplementary material [file 41598_2017_7614_MOESM6_ESM.pdf]

Supplementary Materials for

Formation of a nucleoplasmic reticulum requires *de novo* assembly of nascent phospholipids and shows preferential incorporation of nascent lamins

Authors: Marek M. Drozdz[1], Haibo Jiang[2], Lior Pytowski[1], Chris Grovenor[3], David J. Vaux[1]*

Affiliations:

[1]Sir William Dunn School of Pathology, University of Oxford, Oxford, OX1 3RE, United Kingdom.

[2]Centre for Microscopy, Characterisation and Analysis, The University of Western Australia, 35 Stirling Highway, Crawley, WA 6009, Australia.

[3]Department of Materials, University of Oxford, Oxford, OX1 3PH, United Kingdom.

*Correspondence to: david.vaux@path.ox.ac.uk.

This PDF file includes:

Supplementary Figures 1 to 8

Captions for Supplementary Movies 1 to 5

Other Supplementary Materials for this manuscript include the following:

Supplementary Movies 1 to 5

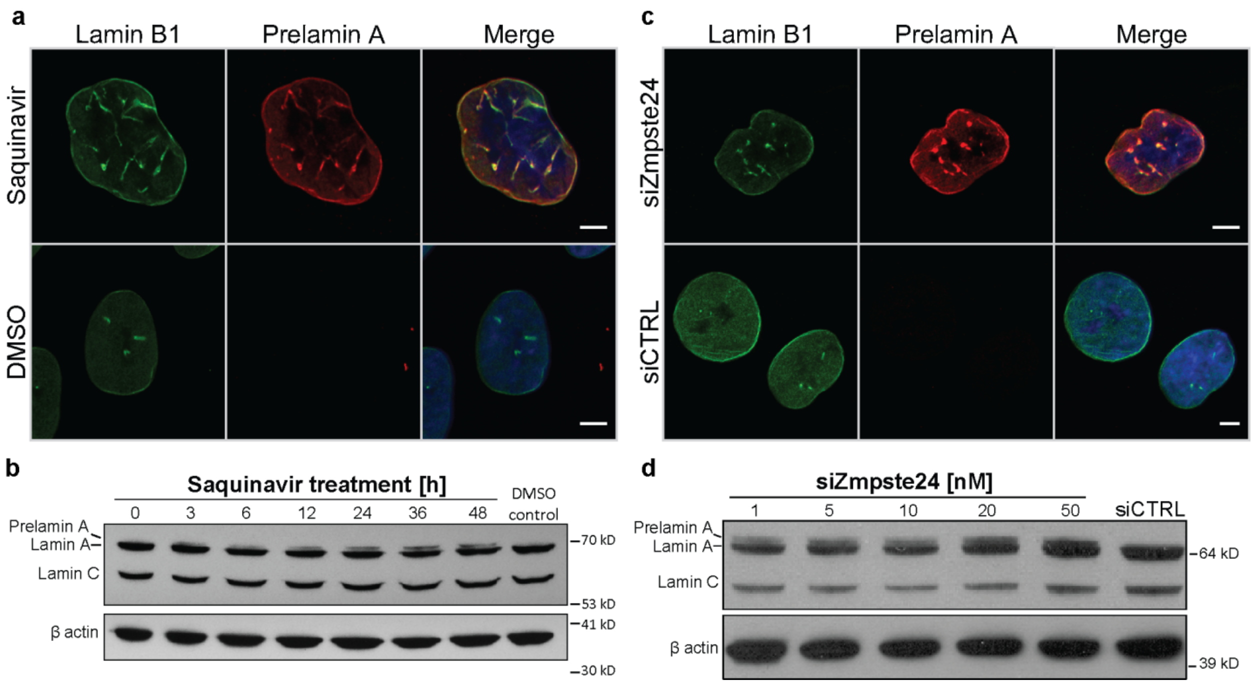

Supplementary Figure 1. Prelamin A accumulation upon saquinavir treatment or siRNA transfection. (**a**) Immunofluorescence microscopy on HDFs treated with either saquinavir or vehicle control (DMSO) over 48 hours period; scale bar, 5 µm. (**b**) Western Blot analysis of whole cell lysates prepared at different time points of saquinavir treatment; DMSO control cells were lysed at 48 hour time point. (**c**) Immunofluorescence microscopy on early passage HDFs transfected with 20 nM Zmpste24 siRNA and imaged 48 hours after transfection; scale bar, 5 µm (**d**) Western Blot analysis of whole cell lysates of early passage HDFs 48 hours post-transfection with indicated concentrations of Zmpste24 siRNA.

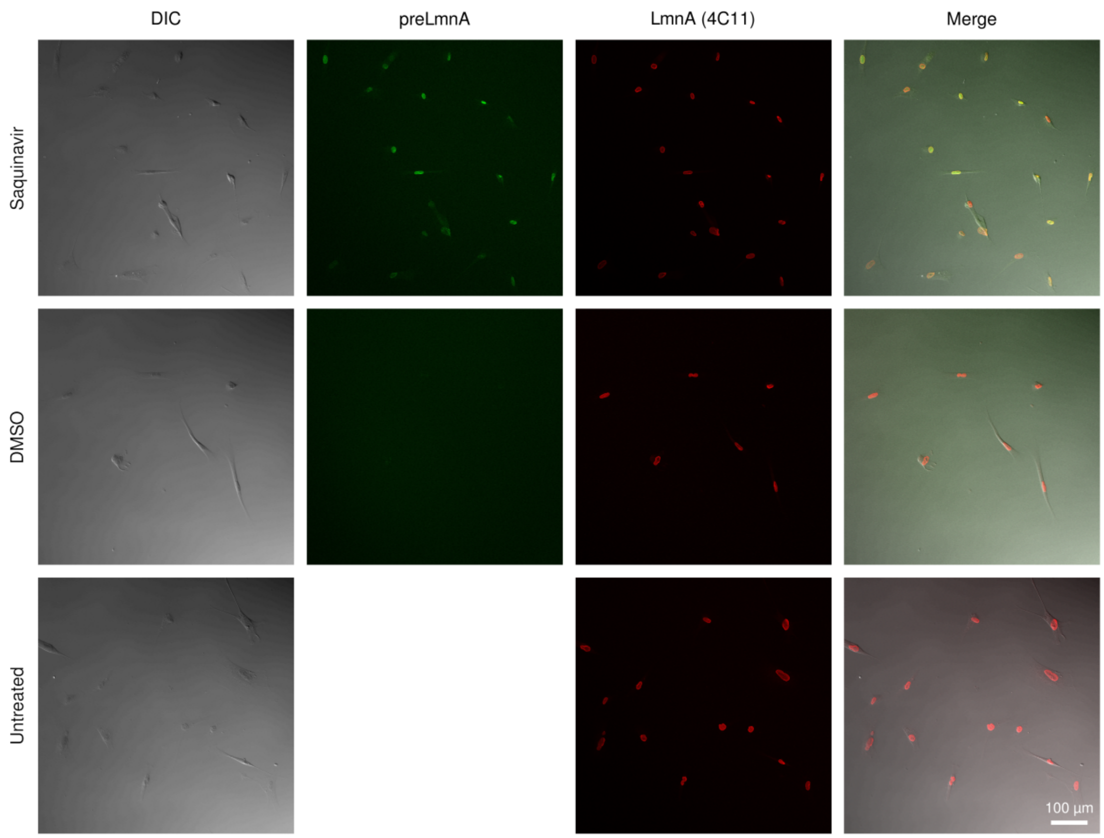

Supplementary Figure 2. Human dermal fibroblasts remain viable after 48 hour saquinavir treatment. A survey field (field of view approximately 750 µm by 750 µm) of human dermal fibroblasts with 48 hour saquinavir treatment, 48 hour DMSO vehicle treatment or no treatment. The panels shown include a DIC image, together with staining for prelamin A or total lamin A. The saquinavir cells have clearly accumulated prelamin A as expected, and have developed complex intranuclear structures as we describe in the manuscript. There is no sign of additional vacuolation, or of evaginated plasma membrane blebs characteristic of apoptotic cells.

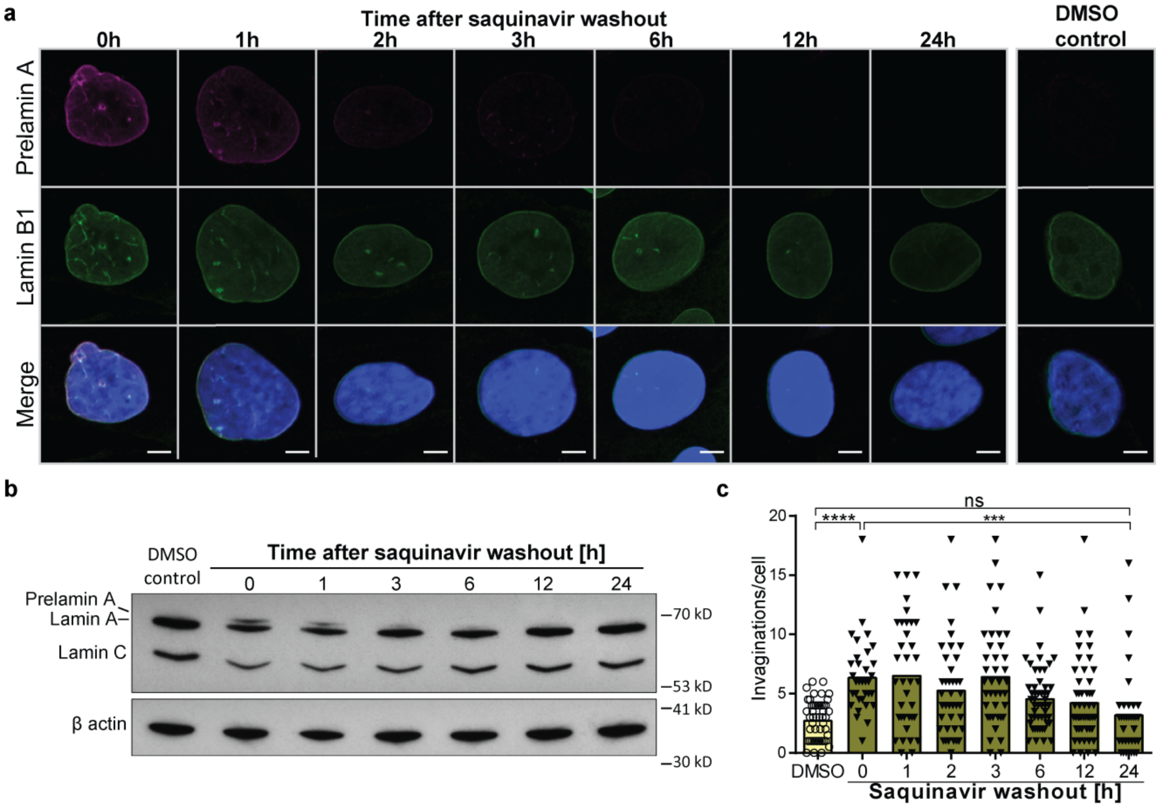

Supplementary Figure 3. Prelamin A is rapidly processed upon saquinavir removal and number of NR invaginations decreases. (**a**) Immunofluorescence microscopy on HDFs upon saquinavir washout; cells were treated with the drug for 48 hours prior to saquinavir withdrawal; images were taken at the indicated time points; scale bar, 5 µm. (**b**) Western Blot analysis of HDF cell lysates prepared at indicated time points after saquinavir removal from culture medium. (**c**) Mean frequency of NR tubules in cells at different time points post saquinavir withdrawal; ***, $p < 0.001$; ****, $p < 0.0001$; ns, p not significant.

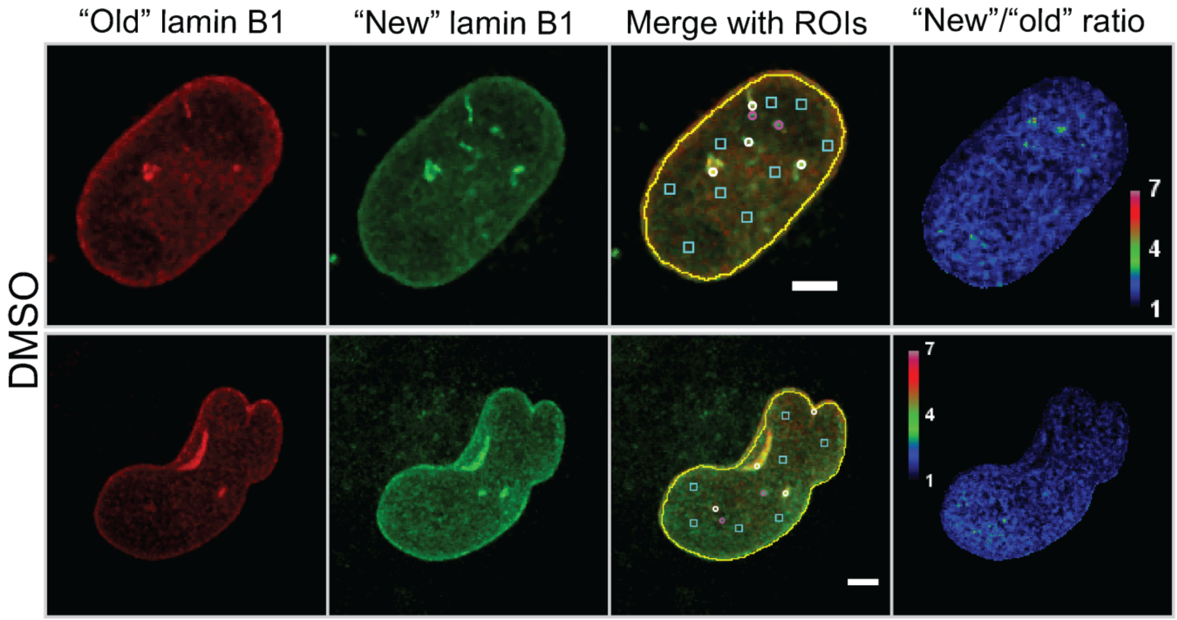

Supplementary Figure 4. MAPLE3 photoconvertible tag allows tracking of nascent lamin B1 delivery to spontaneously forming physiological NR. Examples of HeLa cells exposed to vehicle control (DMSO) and imaged 22-26 hours after complete photoconversion of MAPLE3-lamin B1 that formed NR structures without pathological prelamin A accummulation. "Old" lamin B1 channel corresponds to the pool of lamin B1 existing in the cell immediately after photoconversion, while "new" lamin B1 corresponds to nascent copies of lamin B1 delivered to the nucleus post-conversion. Merge images show Regions of Interest (ROIs) as follows: yellow line, nuclear periphery; white circles, old NR (structures visible in red channel); magenta circles, new NR (structures visible in green channel only); cyan squares, nuclear interior without structures. Ratiometric image comparing pixel intensities of green channel ("new" lamin B1) over red channel ("old" lamin B1). Scale bar, 5 μm.

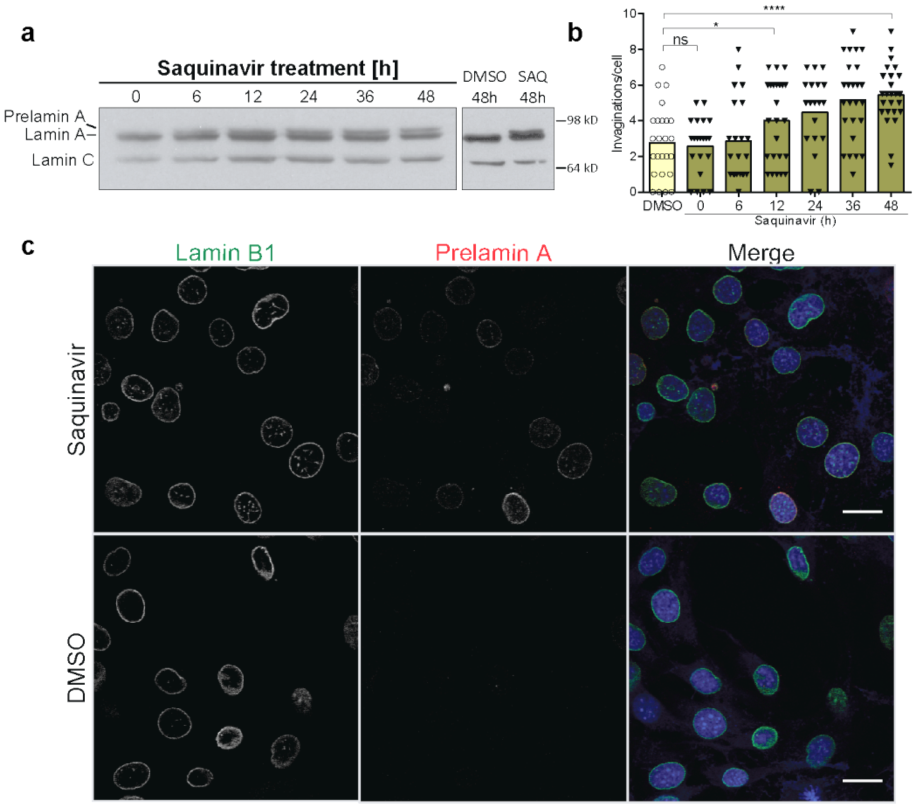

Supplementary Figure 5. Mouse preadipocytes accumulate prelamin A upon saquinavir treatment and form NR. (**a**) Western blot analysis of whole cell lysates prepared at different time points of saquinavir (SAQ) treatment; DMSO control cells were lysed at 48 hour time point. Lamin C was used as a loading control (**b**) Mean frequency of NR tubules in cells accumulating prelamin A under saquinavir treatment (SAQ) at different time points; *, $p < 0.05$; ****, $p < 0.0001$; ns, p not significant. (**c**) Examples of confocal images of cells exposed to either saquinavir or DMSO vehicle for 48 hours; such images were used for quantification of NR abundance identified as bright Lamin B1 foci within the nucleus; scale bar 20 μm.

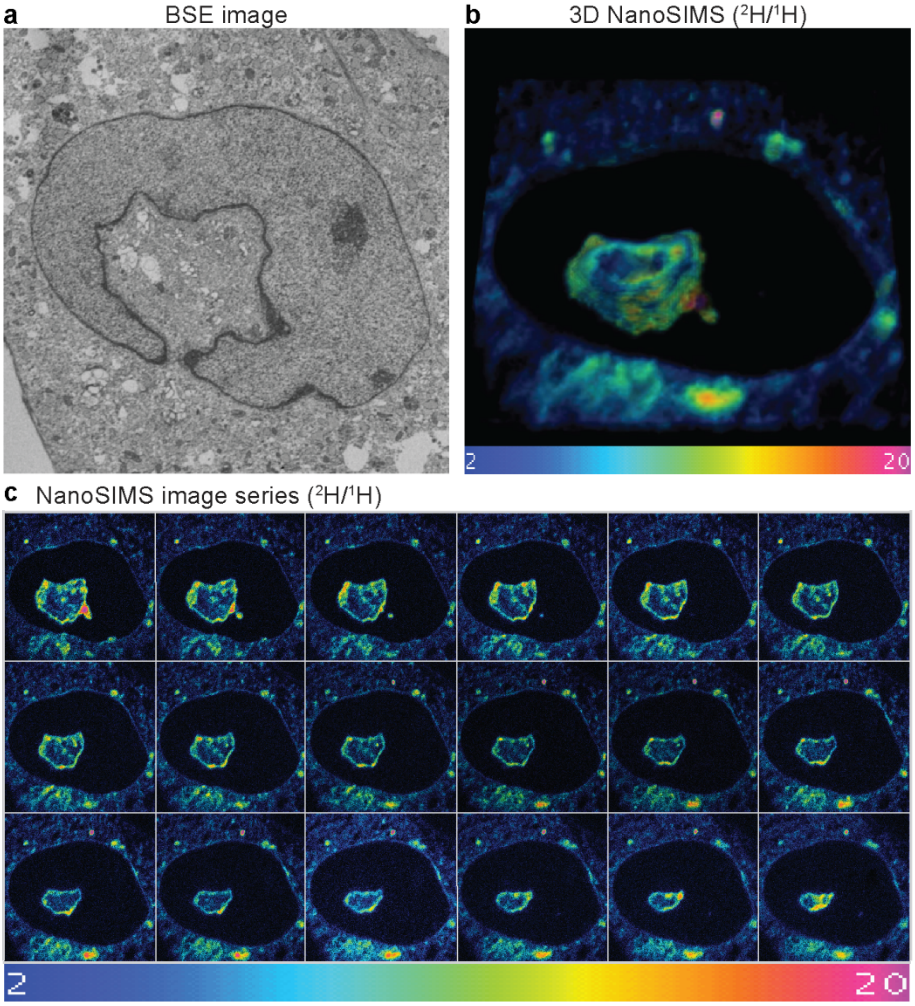

Supplementary Figure 6. 3D NanoSIMS of nascent phospholipid distribution during NR formation. (**a**) Representative backscattered electron (BSE) image of a saquinavir-treated mouse preadipocyte pulsed labelled with deuterated choline. (**b**) 3D reconstruction of NanoSIMS analysis of subsequent layers of the specimen shown in (a). (**c**) Panel of NanoSIMS images used for the 3D reconstruction shown in (b); a single image corresponds to ~10nm specimen thickness. Colour scale of NanoSIMS images 2 – 20 equals 0.02% – 0.2% of $^2H/^1H$ ratio. See corresponding Movie 1.

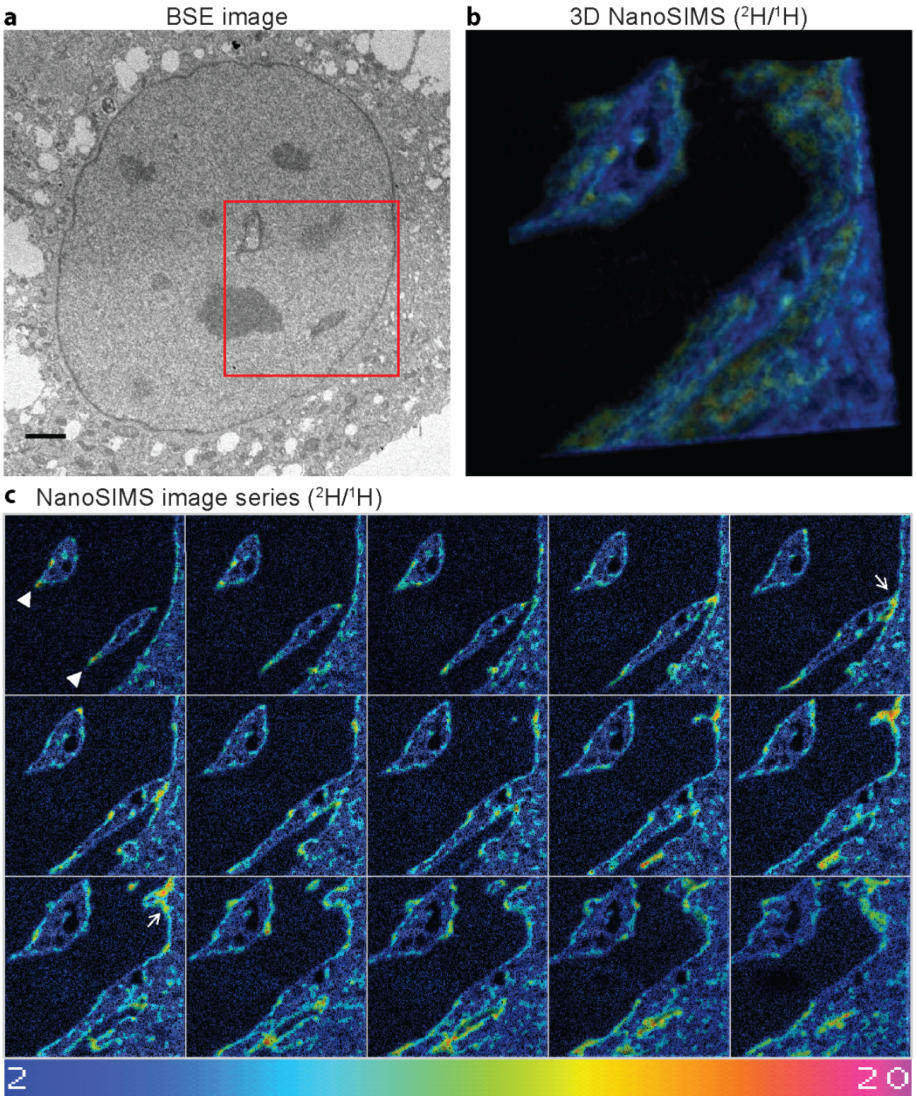

Supplementary Figure 7. 3D NanoSIMS of nascent phospholipid distribution during NR formation. (**a**) Representative backscattered electron (BSE) image of a saquinavir-treated mouse preadipocyte pulsed labelled with deuterated choline; scale bar 2 μm. (**b**) 3D reconstruction of NanoSIMS analysis of subsequent layers of the specimen shown in (a). (**c**) Panel of NanoSIMS images used for the 3D reconstruction shown in (b); examples of tip and base regions of NR tubules are indicated by arrowheads and arrows, correspondingly;

a single image corresponds to ~10nm specimen thickness. See corresponding Movie 4 and 5.

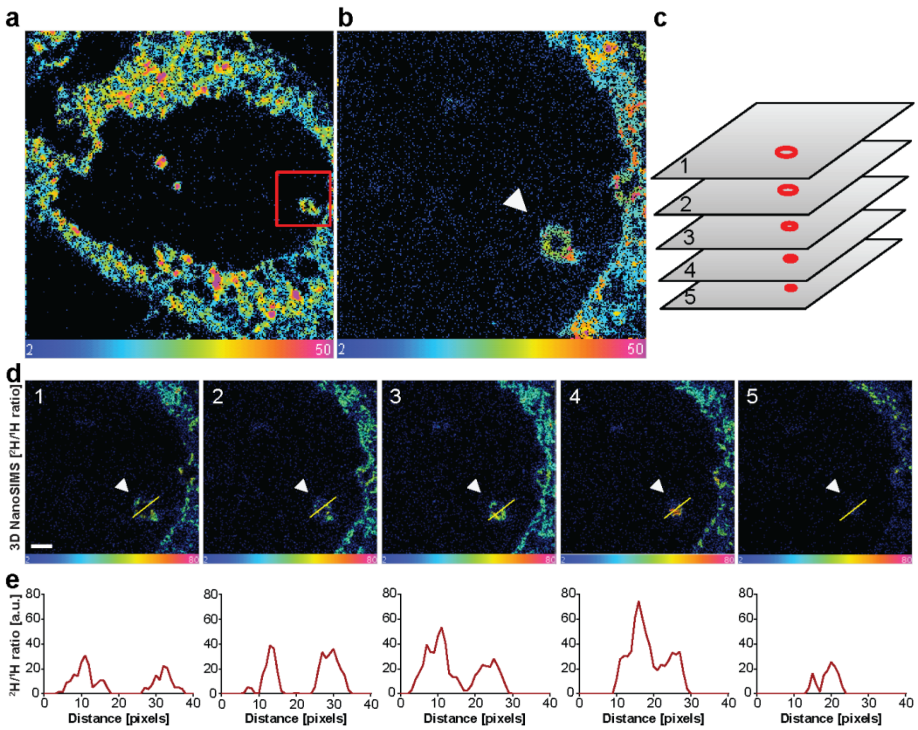

Supplementary Figure 8. Nascent phospholipid distribution along an NR channel. (**a**) NanoSIMS image of nascent lipid distribution in mouse preadipocytes treated with saquinavir and pulse labelled with ^{2}H-stearate. (**b**) Magnification of the region marked with a red rectangle in (a)**.** (**c**) Schematic representation of the orientation of subsequent NanoSIMS images shown in (d). (**d**) NanoSIMS images of consecutive sections showing distribution of nascent phospholipids along the NR tubule shown in (b)**.** (**e**) Graphs corresponding to images shown above in panel (d) that represent the ratio measured along the yellow line, indicating uneven distribution of nascent phospholipids along the NR channel and increased ^{2}H/^{1}H ratio at the tip.

Supplementary Movie 1:

3D NanoSIMS analysis of nascent phospholipid distribution in a mouse preadipocyte nucleus after pulse-chase labelling with deuterated choline. Movie corresponds to Figure 5.

Supplementary Movie 2:

3D NanoSIMS analysis of nascent phospholipid distribution in a mouse preadipocyte nucleus after pulse-chase labelling with deuterated choline. Movie corresponds to Figure 6.

Supplementary Movie 3:

3D NanoSIMS analysis of nascent phospholipid distribution in a mouse preadipocyte nucleus after pulse-chase labelling with deuterated choline. Movie corresponds to Figure 6.

Supplementary Movie 4:

3D NanoSIMS analysis of nascent phospholipid distribution in a mouse preadipocyte nucleus after pulse-chase labelling with deuterated choline. Movie corresponds to Supplementary Figure 7.

Supplementary Movie 5:

3D NanoSIMS analysis of nascent phospholipid distribution in a mouse preadipocyte nucleus after pulse-chase labelling with deuterated choline. Movie corresponds to Supplementary Figure 7.
